# Intravenous contrast use and acute kidney injury: a retrospective study of 1,238 inpatients undergoing computed tomography

**DOI:** 10.1590/0100-3984.2020.0018

**Published:** 2021

**Authors:** Thyago A. Coser, Juliana S. V. Leitão, Betina M. Beltrame, Luciano S. Selistre, Leandro Tasso

**Affiliations:** 1 Universidade de Caxias do Sul (UCS), Caxias do Sul, RS, Brazil.

**Keywords:** Acute kidney injury, Tomography, X-ray computed, Contrast media/adverse effects, Contrast media/administration & dosage, Iodine radioisotopes, Creatinine/blood, Lesão renal aguda, Tomografia computadorizada, Meios de contraste/efeitos adversos, Meios de contraste/administração & dosagem, Radioisótopos do iodo, Creatinina/sangue.

## Abstract

**Objective:**

To determine the incidence of nephropathy induced by intravenous contrast in hospitalized patients undergoing computed tomography (CT).

**Materials and Methods:**

This was a retrospective cohort study involving 1,238 patients who underwent CT with or without intravenous administration of a contrast agent (iopromide). The primary outcome measure was acute kidney injury (AKI), as defined by the traditional criteria-an absolute or relative increase in serum creatinine (SCr) ≥ 0.5 mg/dL or ≥ 25% over baseline, respectively, at 2-3 days after contrast administration-and the newer, Kidney Disease: Improving Global Outcomes (KDIGO) criteria-an absolute or relative increase in SCr ≥ 0.3 mg/dL or ≥ 50% over baseline, respectively, at 2-7 days after contrast administration.

**Results:**

The overall incidence of AKI was 11.52% when the KDIGO criteria were applied. Univariate logistic regression demonstrated a significant association between an absolute post-CT increase in SCr ≥ 0.5 mg/dL and AKI, although that association did not retain significance in the multivariate analysis. Multivariate logistic regression initially found an association between an absolute post-CT increase in SCr ≥ 0.3 mg/dL and advanced age, although that association was not maintained after correction. We found no association between AKI and the risk factors evaluated.

**Conclusion:**

We identified no criteria for contrast-induced nephropathy after CT; nor did we find AKI to be associated with the classical risk factors.

## Abstract (pt)

**Objetivo:**

Determinar a incidência de nefropatia induzida por contraste intravenoso em pacientes hospitalizados submetidos a tomografia computadorizada (TC).

**Materiais e Métodos:**

Estudo de coorte retrospectivo que alocou 1.238 pacientes submetidos a TC sem ou com contraste (iopromida). O desfecho primário foi nefropatia induzida por contraste, definida pelo critério antigo - aumento absoluto ou relativo na creatinina sérica (SCr) ≥ 0,5 mg/dL ou ≥ 25%, respectivamente, durante 2-3 dias após a administração -, e o novo, Kidney Disease Improving Global Outcomes (KDIGO) - aumento absoluto ou relativo na SCr ≥ 0,3 mg/dL ou ≥ 50%, respectivamente, durante 2-7 dias após a administração.

**Resultados:**

A incidência de lesão renal aguda foi de 11,52% aplicando os critérios KDIGO. A regressão logística univariada demonstrou significância relacionada à associação entre aumento absoluto da SCr ≥ 0,5 mg/dL após TC e lesão renal aguda. A regressão logística multivariada encontrou, inicialmente, associação entre aumento absoluto da SCr ≥ 0,3 mg/dL após TC e idade avançada, mas a associação não foi mantida após correção. Não foi encontrada associação entre lesão renal aguda e os fatores de risco avaliados.

**Conclusão:**

Não foram encontrados critérios para nefropatia induzida por contraste após TC ou associação de lesão renal aguda com fatores de risco clássicos.

## INTRODUCTION

Intravenous administration of iodinated contrast media is widely used during computed tomography (CT) examinations because it can increase diagnostic accuracy^(1)^. However, the use of such media is not risk-free, because they are nephrotoxic and can cause renal dysfunction^(2)^. Medical societies initially defined contrast-induced nephropathy (CIN), manifesting as acute kidney injury (AKI), on the basis of the following criteria: an absolute or relative increase in serum creatinine (SCr) ≥ 0.5 mg/dL or ≥ 25% over baseline, respectively, at 2-3 days after contrast administration^(3)^. After the publication of the Kidney Disease: Improving Global Outcomes (KDIGO) study^(4)^, the criteria for CIN were changed to an absolute or relative increase in SCr ≥ 0.3 mg/dL or ≥ 50% over baseline, respectively, at 2-7 days after contrast administration.

In animal models, the pathogenesis of CIN has been shown to consist of classic acute tubular necrosis, secondary to vasoconstriction, accompanied by medullar hypoxia and, thereafter, tubular cell cytotoxicity. Vascular damage occurs due to a reduction in nitric oxide levels, together with increases in endothelin and adenosine in the bloodstream^(5)^.

With an average incidence of 11%, CIN is recognized as the third leading cause of AKI in hospitalized patients^(6)^. However, the reported incidence ranges from 1% and 50%, depending on the characteristics of the population studied and whether intra-arterial or intravenous contrast is used^(7-10)^. Among patients receiving intravenous contrast alone, the reported incidence of CIN can vary from 2.5% to 12%^(7-10)^. The classical risk factors for CIN are female gender, advanced age, diabetes mellitus, cardiovascular disease, infectious processes, chronic kidney disease (CKD), and nephrotoxic drug use^(4-11)^.

Most studies in the literature address CIN after the administration of intra-arterial contrast, few having evaluated it after intravenous contrast administration. In view of the small number of studies of the latter topic^(1,11-18)^, we carried out the present study to evaluate the relationship between intravenous contrast administration and CIN in hospitalized patients, as well as to determine the influence that classical risk factors have on that relationship.

## MATERIALS AND METHODS

This was a single-center retrospective cohort study, conducted between August 1, 2010 and April 30, 2017 at the General Hospital of Caxias do Sul, located in the city of Caxias do Sul, Brazil, which is a regional referral center serving a population of 1.5 million people. The study was approved by the Research Ethics Committee of the University of Caxias do Sul (Reference no. 1880766). All of the authors signed a confidentiality agreement to ensure the anonymity of the data obtained from the electronic medical records of the hospital.

Between August 1, 2010 and April 30, 2017, a total of 129,205 patients were admitted to the General Hospital of Caxias do Sul, and 5,159 inpatients underwent CT. We analyzed the electronic medical records of those 5,159 patients, comparing the incidence of CIN between those who received intravenous contrast and those who did not receive any contrast.

The SCr level was tracked by isotope dilution mass spectrometry. Contrast administration was performed in accordance with the institutional protocol, which calls for intravenous infusion of the non-ionic contrast agent iopromide (Ultravist 300; Bayer AG, Berlin, Germany) at a concentration of 623 mg/mL (equivalent to 300 mg/mL of iodine), with an osmolality of 0.59 mOsm/kg.

The following inclusion criteria were applied: having been admitted to the hospital; having undergone CT, with or without contrast enhancement; having been ≥ 18 years of age on the date of the CT; having undergone SCr determination at baseline and at 48 h after CT; having had stable renal function during the 3 months preceding the CT examination; and having had a baseline SCr of 0.4-4.0 mg/dL. Patients who had not shown an absolute increase in SCr ≥ 0.3 mg/dL over a 48-h period, a relative increase in SCr ≥ 1.5 times the baseline value over a 7-day period, or diuresis < 0.5 mL/kg/h over a 6-h period were categorized as having stable renal function^(2)^. Patients on dialysis were excluded, as were those in whom contrast media had been administered for other radiological purposes in the 48 h prior to the CT examination. On the basis of the study criteria, 3,921 patients were excluded. Therefore, the final sample comprised 1,238 patients, of whom 642 (51.85%) underwent contrast-enhanced CT and 596 (48.15%) underwent unenhanced CT.

To look for correlations with CIN, we analyzed the following explanatory variables: age; gender; advanced age (≥ 65 years); diabetes mellitus, as defined in the International Diabetes Federation guidelines^(19)^; cardiovascular disease, as defined in the New York Heart Association Functional Classification^(20)^; sepsis, as defined by the Latin American Sepsis Institute^(21)^; CKD, diagnosed in accordance with the 2012 KDIGO criteria^(4)^; obesity, defined as a body mass index ≥ 30 kg/m^2^, as established by the World Health Organization^(22)^; the previous use of nephrotoxic drugs, including angiotensin-converting enzyme inhibitors, angiotensin receptor blockers, nonsteroidal anti-inflammatory drugs, aminoglycosides, and others; and the use of preventive measures (crystalloids, sodium bicarbonate, or N-acetylcysteine). The primary outcome measure was CIN. The criteria for AKI were increases in SCr at 48 h after CT, based on the KDIGO criteria (0.3 mg/dL or 50% over baseline) or on the traditional criteria (0.5 mg/dL or 25% over baseline). In addition to evaluating the risk factors for CIN listed above, we attempted to determine whether the contrast volume affected the post-CT level of SCr and whether the adoption of preventive measures reduced the incidence of CIN, the secondary outcome measures therefore being the post-CT level of SCr and the incidence of CIN.

### Statistical modeling

Continuous variables are expressed as mean and standard deviation, whereas categorical variables are presented as absolute and relative values. Differences in proportions were assessed by the chi-square test, when indicated. The normality of the distribution of continuous variables was calculated with the Shapiro-Wilk test. Student's t tests were used for comparisons between continuous variables. The variables that differed between patients with and without CIN were analyzed by stepwise logistic regression with backward selection, and the Wald test was used in order to look for associations with the occurrence of CIN. To quantify the effects, we calculated odds ratios (ORs) and the respective 95% confidence intervals (95% CIs).

The statistical analysis was performed with the software R for Windows, version 3.3.2 (R Development Core Team-www.r-project.org). We aimed to avoid information bias by robust selection and statistical analysis.

## RESULTS

On the basis of the study criteria, we excluded a total of 3,921 patients. Therefore, the final sample comprised 1,238 patients, corresponding to 24.0% of the eligible patients. Of those 1,238 patients, 642 (51.85%) underwent contrast-enhanced CT and 596 (48.15%) underwent unenhanced CT.

The demographic, clinical, and biochemical characteristics of the sample are presented in Table 1. Of the 1,238 patients, 723 (58.41%) were men and 515 (41.59%) were women. The mean age was 60.8 ± 16.1 years in the sample as a whole, being significantly higher in the patients who underwent unenhanced CT than in those who underwent contrast-enhanced CT (63.5 ± 16.08 vs. 59.3 ± 15.8 years; *p* < 0.01). Comparing the patients who underwent unenhanced CT with those who underwent contrast-enhanced CT, we found that 40.03% and 54.53%, respectively, were ≥ 65 years of age (*p* < 0.01); 16.66% and 23.82%, respectively, had diabetes mellitus (*p* = 0.013); 25.54% and 46.30%, respectively, had CKD (*p* < 0.01); and 19.62% and 11.74%, respectively, had been submitted to preventive measures (*p* < 0.01). The other variables related to the characteristics of the patients did not differ between those who underwent unenhanced CT and those who underwent contrast-enhanced CT. As can also be seen in Table 1, the median SCr level was lower in the patients who underwent contrast-enhanced CT than in those who underwent unenhanced CT, at baseline (0.8 mg/dL vs. 1.1 mg/dL; *p* < 0.01) and at 48 h after CT (0.8 mg/dL vs. 1.05 mg/dL; *p* < 0.01).

**Table 1:** Demographic, clinical, and biochemical characteristics of patients undergoing CT with and without intravenous contrast administration.

Characteristic	All (n = 1,238)	Contrast-enhanced (n = 642)	Unenhanced (n = 596)	*P*
Age (years), mean ± SD	60.8 ± 16.1	59.3 ± 15.8	63.5 ± 16.08	< 0.01
Female gender, n (%)	515 (41.59)	248 (38.62)	267 (44.79)	1.00
Advanced age, n (%)	582 (47.01)	257 (40.03)	325 (54.53)	< 0.01
Diabetes mellitus, n (%)	249 (20.11)	107 (16.66)	142 (23.82)	0.013
Cardiovascular disease, n (%)	109 (8.80)	36 (5.60)	73 (12.24)	0.129
Infection, n (%)	201 (16.23)	107 (16.66)	94 (15.77)	0.726
CKD, n (%)	440 (35.54)	164 (25.54)	276 (46.30)	< 0.01
Obesity, n (%)	95 (7.67)	52 (8.09)	43 (7.21)	1.00
Previous nephrotoxic drug use, n (%)	712 (57.51)	366 (57.01)	348 (58.38)	0.448
CIN preventive measures, n (%)	196 (15.83)	126 (19.62)	70 (11.74)	< 0.01
Baseline SCr (mg/dL), median (IQR)	1.27 (0.6-1.4)	0.8 (0.62-1.2)	1.1 (0.8-1.6)	< 0.01
48 h post-CT SCr (mg/dL), median (IQR)	1.30 (0.6-1.4)	0.8 (0.6-1.1)	1.05 (0.7-1.6)	< 0.01

IQR, interquartile range

The univariate logistic regression showed that an absolute post-CT increase in SCr ≥ 0.5 mg/dL correlated significantly with the incidence of AKI (OR = 5.4; 95% CI: 1.45-20.15; *p* = 0.02). However, the incidence of AKI was not found to correlate significantly with an absolute post-CT increase in SCr ≥ 0.3 mg/dL or with a relative post-CT increase in SCr ≥ 25% or ≥ 50% over baseline (Table 2). Applying the KDIGO criteria, we found that the incidence of AKI among the patients who underwent contrast-enhanced CT was 11.52% when based on the absolute post-CT increase in SCr and 7.47% when based on the relative post-CT increase in SCr (Table 3).

**Table 2:** Increase in serum creatinine after CT.

Post-CT increase in SCr	OR	95% CI	*P*
≥ 0.3 mg/dL	1.15	0.62-2.15	0.65
≥ 0.5 mg/dL	5.4	1.45-20.15	0.02
≥ 25% over baseline	2.6	0.90-7.4	0.07
≥ 50% over baseline	3.5	0.92-13.1	0.06

**Table 3:** Incidence of AKI after CT, with and without intravenous contrast administration, by post-CT increase in SCr.

	AKI after CT		
Post-CT increase in SCr	All (n = 1,238)	Contrast-enhanced (n = 642)	Unenhanced (n = 596)
≥ 0.3 mg/dL, n (%)	197 (15.91)	74 (11.52)	123 (20.63)
≥ 0.5 mg/dL, n (%)	128 (10.33)	41 (6.38)	87 (14.59)
≥ 25% over baseline, n (%)	229 (18.49)	102 (15.88)	127 (21.30)
≥ 50% over baseline, n (%)	113 (9.12)	48 (7.47)	65 (10.90)

Table 4 shows the results of the multivariate logistic regression of the explanatory variables in relation to the absolute post-CT increases in SCr ≥ 0.3 mg/dL and 0.5 mg/dL, as well as in relation to the relative post-CT increases in SCr ≥ 25% and ≥ 50% over baseline. Another point worth noting is that the multivariate analysis revealed that AKI showed a significant positive association only with an absolute post-CT increase in SCr ≥ 0.3 mg/dL and only in the elderly patients (OR: 1.77; 95% CI: 1.01-3.11; *p* = 0.05), although that association did not retain its significance after correction.

**Table 4:** Risk factors for AKI after CT, by post-CT increase in SCr.

Variable	OR	95% CI	*P*
Post-CT increase in SCr ≥ 0.3 mg/dL			
Risk factors			
Contrast use	0.72	0.42-1.24	0.24
Female gender	1.22	0.70-2.11	0.48
Advanced age	1.77	1.01-3.11	0.05
Diabetes mellitus	0.63	0.31-1.28	0.20
Cardiovascular disease	0.78	0.25-2.41	0.67
Infection	1.40	0.72-2.72	0.32
CKD	1.25	0.69-2.24	0.46
Obesity	1.26	0.64-2.47	0.51
Previous nephrotoxic drug use	0.88	0.51-1.51	0.64
Post-CT increase in SCr ≥ 0.5 mg/dL			
Risk factors			
Contrast use	0.82	0.41-1.64	0.58
Female gender	1.20	0.59-2.45	0.61
Advanced age	1.37	0.66-2.83	0.40
Diabetes mellitus	0.48	0.18-1.26	0.14
Cardiovascular disease	0.61	0.13-2.81	0.53
Infection	1.61	0.72-3.61	0.25
CKD	1.57	0.74-3.34	0.24
Obesity	2.18	0.98-4.83	0.06
Previous nephrotoxic drug use	1.14	0.57-2.30	0.71
Post-CT increase in SCr ≥ 25% over baseline			
Risk factors			
Contrast use	1.08	0.65-1.79	0.76
Female gender	1.28	0.76-2.14	0.35
Advanced age	1.38	0.82-2.34	0.23
Diabetes mellitus	0.76	0.39-1.48	0.42
Cardiovascular disease	1.04	0.37-2.91	0.94
Infection	1.65	0.90-3.03	0.10
CKD	0.69	0.38-1.24	0.22
Obesity	1.08	0.57-2.04	0.82
Previous nephrotoxic drug use	0.87	0.53-1.43	0.59
Post-CT increase in SCr ≥ 50% over baseline			
Risk factors			
Contrast use	0.87	0.43-1.78	0.71
Female gender	0.87	0.43-1.77	0.70
Advanced age	1.53	0.74-3.19	0.25
Diabetes mellitus	0.59	0.22-1.55	0.28
Cardiovascular disease	0.34	0.04-2.63	0.30
Infection	1.50	0.64-3.48	0.35
CKD	0.72	0.32-1.66	0.45
Obesity	2.12	0.96-4.68	0.06
Previous nephrotoxic drug use	1.13	0.55-2.29	0.74

## DISCUSSION

Initial reports on CIN inferred a strong association between intra-arterial contrast use (for angiographic procedures) and AKI. Because of that relevant incidence, other studies were conducted in order to determine whether CT examinations performed with intravenous contrast were associated with AKI^(1,11-18)^.

In the present study, we expected that some patients would develop CIN within the first 48 h after contrast injection, especially because we used a contrast agent with an osmolality of 0.59 mOsm/kg (formerly referred to as "low osmolar contrast") rather than an iso-osmolar contrast agent. However, we did not observe a relevant increase in SCr in the patients undergoing procedures involving the use of intravenous contrast. Because this was a retrospective study, one possible explanation for this result is that the medical indications for intravenous contrast administration were not homogeneous, having been set by the institutional protocol, although the clinical characteristics, risk factors, and preventive measures were probably taken into account. This result is corroborated by evidence in recent studies showing that the incidence of CIN after intravenous contrast administration is relatively low^(1,14-17)^. However, many of those were also retrospective studies. The authors of those studies concluded that intravenous contrast administration is not a risk factor for AKI, even in patients with impaired renal function or with comorbidities that can predispose to nephrotoxicity^(1,15-17)^. Nevertheless, other authors have concluded that intravenous contrast administration is a risk factor for AKI in patients with CKD and diabetes mellitus^(7,9,10,18)^.

In the present study, the incidence of AKI was 11.52% when we applied the KDIGO criteria, although it was not found to be associated with intravenous contrast use. In two meta-analyses, evaluating 42 and 40 articles, respectively, the respective incidence of CIN after intravenous contrast administration was 4.96% and 6.4%^(9,10)^. In another retrospective study, involving 126 patients with a glomerular filtration rate (GFR) < 60 mL/min/1.73 m^2^, the incidence of CIN was found to be 5.1%^(8)^. In a heterogeneous prospective cohort of patients undergoing contrast-enhanced CT, 11% developed AKI consistent with CIN^(2)^.

In the univariate logistic regression, we found a statistically significant association between an absolute post-CT increase in SCr ≥ 0.5 mg/dL and AKI. However, that association did not retain its significance after adjustment for the use of contrast and risk factors in the multivariate logistic regression. Similarly, the positive association between an absolute post-CT increase in SCr ≥ 0.3 mg/dL and advanced age did not retain its significance after correction in the multivariate analysis. We found no other associations with classical risk factors, thus corroborating the results of previous studies^(1,11,14-17)^.

Our study has some limitations. Because it was a retrospective study in which data were collected from electronic medical records, some relevant information was missing. Consequently, we were unable to analyze the association between contrast volume and post-CT SCr, because patient height was not noted in 943 (76.17%) of the medical records reviewed. That underscores the need to measure and register such data in medical records. Despite the missing data and the fact that the contrast dose was not adjusted to the specific needs of the individual patients, we found no association between contrast use and AKI, thus supporting the hypothesis that the use of contrast during CT does not induce CIN. Another potential limitation is the fact that the proportion of patients who were elderly was significantly higher among the patients who underwent unenhanced CT, as was that of those who had diabetes mellitus and that of those who had CKD. That was probably because physicians had contraindicated the use of contrast on the basis of the medical history of those patients. In contrast, preventive measures were used at a significantly higher rate in the patients who underwent contrast-enhanced CT. That could explain the lack of differences between the two groups in terms of the incidence of CIN. Many studies have used propensity matching analysis to avoid these differences among groups in retrospective studies^(23)^.

The Royal College of Radiology recommends the infusion of 0.9% saline solution for patients at risk for CIN^(24)^. A recent randomized clinical trial showed that intravenous hydration did not reduce the incidence of CIN for patients with a GFR of 30-59 mL/min/1.73 m^2^, suggesting that intravenous rehydration should be reserved for patients with a GFR < 30 mL/min/1.73 m^2^^(25)^. Another study suggested that CIN preventive measures were not effective in a population of patients undergoing CT and stratified into subgroups according to baseline GFR: ≥ 90, 60-89, 30-59, and < 30 mL/min/1.73 m^2^^(13)^. In our investigation, CIN was not found. Therefore, the value of preventive measures as a protective factor for AKI after intravenous contrast use could not be evaluated.

In our sample, there was an increase in SCr at 48 h after CT in both groups of patients (those who received intravenous contrast and those who did not). Therefore, SCr was not associated with the use of contrast; nor was it associated with any of the classical risk factors studied. Many studies have shown that there is day-to-day variability in creatinine levels in hospitalized patients, which can be related to the underlying disease, to the indication for the CT, or even to iatrogenic causes. Although further studies are needed in order to identify possible associated factors, the risk of CIN has been overestimated in clinical practice^(26)^. The results of the present study are valid for our hospital but cannot be generalized to other institutions.

## CONCLUSION

In our study, the incidence of AKI was 11.52%. We identified no other criteria for CIN after CT. We also found no association between AKI and classical risk factors. This corroborates the current literature, in which the incidence of CIN after intravenous contrast administration seems to be lower than previously thought.
